# Unmasking the Hypoxia Landscape in Cervical Cancer: S100A2 and Its Implication for Immunotherapy Resistance

**DOI:** 10.1007/s43032-023-01336-3

**Published:** 2023-08-31

**Authors:** Mohamed Laban, Xi Chen, Bing Guo

**Affiliations:** 1 Gynecologic Oncology Unit, Department of Obstetrics and Gynecology, Ain Shams University, 38 Ramsis St., Abbasseya, 11591 Cairo Egypt; 2 National Cancer Institute, National Institutes of Health, Bethesda, MD USA; 3 University of Maryland School of Medicine, Baltimore, MD USA

**Keywords:** Hypoxia, Immunotherapy, S100A1, PD-L1

## Abstract

Cervical cancer is the fourth most common cancer in women worldwide and typically diagnosed between the ages of 35 and 44. Despite the death rate declining 1% each year since the 2000s, the 5-year survival of late stage remains lower than 20%. This emphasizes the urgency to keep exploring cervical cancer cell survival factors and identifying new prognostic markers. In this issue of Reproductive Sciences, Yang et al. stratified hypoxia subtype by analyzing 200 hypoxia-related genes in TCGA database and observed patient overall survival, hypoxic, transcriptome, genomics, and immunological characteristics vary among these hypoxia subtypes and created a hypoxia score which successfully stratified patient by predicting clinical outcomes and response to immunotherapy. Simultaneously, a hypoxia mediator (S100A2) associated with an aggressive cervical cancer phenotype is identified. We reviewed similar work on S100A2 and hypoxia-mediated multidrug resistance and highlighted the values added by this study. Future work could focus on unraveling the direct link between S100A2 and immunotherapy resistance.

Cervical cancer is the fourth frequent cancer among women worldwide between the ages of 35 and 44. Globally, it is estimated that more than 6 million women suffered from cervical cancer. Although early screening and increased awareness of risk factors have resulted in a 1% annual decline in the death rate since the 2000s, the five-year survival rate for late stage remains alarmingly low, at under 20%. It is necessary to explore cervical cancer cell survival factors and identifying more prognostic markers.

Research has established a close link between hypoxia and multidrug resistance in cancer treatment, through mechanisms like enlargement of cancer stem cell populations and creation of a pro-metastasis tumor microenvironment [1]. A goal of this study from Yang and colleagues [2] is to explore the hypoxia landscape in cervical cancer. They stratified hypoxia subtype by analyzing 200 hypoxia-related genes in TCGA database and observed variability in patient overall survival, hypoxic, transcriptome, genomics, and immunological characteristics across these hypoxia subtypes. Meanwhile, they also developed a “hypoxia score,” which successfully stratified patients by predicting clinical outcomes and responses to immunotherapy. Considering the high frequency of chemoresistance that is observed in late stage of cervical cancer, immunotherapy is used in combination with chemotherapy in patients with recurrent or metastatic cervical cancer. A notable example is the immune checkpoint inhibitor pembrolizumab (Keytruda), a PD-L1 inhibitor approved to treat cervical cancer that has recurred or spread during or after chemotherapy, particularly in cases where the tumor expresses PD-L1. Therefore, the “hypoxia score” holds significant clinical implications and could potentially guide the design of more personalized future treatment strategies. By mapping patient genomic data into hypoxia landscape, treatment strategies might become more efficient and advanced, building upon the distinct characteristics of patients based on hypoxia subtypes and “hypoxia score.”

One strength of this study lies in its experimental verification of the proposed hypoxia mediator (S100A2). Though high S1002A2 may predict poor prognosis in endometrial carcinoma [3], the functions of S100A2 in cervical cancer have not been well established yet. Using single cell RNA sequencing technique, S100A2 was identified as an immunosuppressive factor that is increased responding to hypoxia and mediates PD-L1 expression. Reduction of S100A2 level is associated with reduced cervical cancer cell phenotype, evidenced by reduced cell proliferative, invasion, and migration, and that phenotype reduction mediated by S100A2 knockdown antagonizes hypoxia action, suggesting S100A2 could be a crucial hypoxia mediator that drives cervical cancer phenotype and immunotherapy resistance by regulating PD-L1. As less than 60% of cervical cancers express PD-L1 [4], the mechanism of S100A2 upregulation driving PD-L1 expression and leading to immunotherapy resistance necessitates further exploration, specifically whether S100A2 is associated with patient outcomes linked to PD-L1 expression. From this study, it remains unclear whether S100A2 can stratify overall survival as well as distal metastasis free survival in patients based on S100A2 expression. This information forms a crucial basis for exploring the potential of S100A2-based therapeutic strategies. Meanwhile the application of immunotherapy is currently limited to patients with recurrent or metastatic cervical cancer so if the authors adjust their study objects to the patient group requiring immunotherapy, the results from this study would be more meaningful. Additionally, this study did not provide directly evidence showing S100A2 is associated with immunotherapy resistance. This gap could be filled by future experiments examining whether the overexpression of S100A2 replicates the effects of hypoxia in driving an aggressive cervical cancer phenotype and if its overexpression counteracts the action of PD-L1 inhibitor pembrolizumab in reducing cancer phenotype. Preclinical in vivo studies represent the gold standard for demonstrating a candidate’s potential role in driving immunotherapy resistance. Using cervical cancer metastasis animal model, whether S100A2 is a key cervical cancer metastasis driver and if the level of S100A2 affects the efficacy of pembrolizumab could be directly revealed.

Growing evidence points to the benefits of immunotherapy over traditional treatment types. One hypothesis suggests the reduced efficacy of immunotherapy in solid cancers, as compared to hematologic malignancies, may be due to the hypoxic conditions present in solid tumors [5]. This study by Yang et al. is well-designed and structured. It seamlessly combines data-based prediction in patient prognosis with experimental verification of a potential hypoxia mediator (S100A2) that is potentially important in driving immunotherapy resistance. Despite its limitations, it provides a strong foundation for future research. Future studies are more encouraged to focus on specific cervical cancer patient populations, such as those in advanced stage of cervical cancers or with recurrent cervical cancers. A key emphasis could also present direct evidence of the association between S100A2 and immunotherapy resistance. This could involve exploration of whether overexpressed S100A2 antagonizes PD-L1 inhibitor action in vitro and in cervical cancer metastasis animal model. Meanwhile, the study of the mechanism by which S100A2 regulates PD-L1 is also of considerable value. Immunotherapy holds promise for patients in late stages or with recurrent cancers who have shown limited response to chemotherapy which make ongoing studies to identify further immunosuppressive factors are of great importance. This study may direct future research focused on hypoxia-driven immunotherapy resistance.

## Data Availability

The data and material that support the findings of this commentary are available from the corresponding author upon request.
